# Associations between maternal dietary intake and nutritional status with fetal growth at 14 to 26 weeks gestation: a cross- sectional study

**DOI:** 10.1186/s40795-024-00885-3

**Published:** 2024-05-23

**Authors:** Margaret Kiiza Kabahenda, Barbara J. Stoecker

**Affiliations:** 1 Department of Food Technology and Nutrition, College of Agricultural and Environmental Sciences, Makerere University, Kampala, Uganda; 2 Department of Nutritional Sciences, Oklahoma State University, Stillwater, OK USA

**Keywords:** Pregnancy, Dietary intake, Diet quality, Fetal growth, Symphysis fundal height, Anemia, Mid-upper arm circumference or MUAC, Women’s health, Uganda, Africa

## Abstract

**Background:**

Maternal undernutrition during pregnancy is currently estimated at 23.5% in Africa, which is worrying given the negative impacts of malnutrition on maternal and fetal birth outcomes. The current study aimed at characterizing the associations of maternal dietary intake and nutritional status with fetal growth at 14–26 weeks gestation. It was hypothesized that maternal dietary intake was positively associated with maternal nutritional status and fetal growth both in early and late pregnancy.

**Methods:**

This was a cross-sectional survey of 870 pregnant women in mid-western Uganda conducted in August 2013. Data were collected on women’s dietary intake (indicated by women’s dietary diversity and the diet quality score) and nutritional status (indicated by hemoglobin level and mid-upper arm circumference) at 14–26 weeks gestation. Fetal growth was determined by symphysis-fundal height Z-scores processed using the INTERGROWTH-21st calculator. Associations between maternal dietary intake and nutritional status with fetal growth were determined using correlations and chi-square tests.

**Results:**

Overall, only 25% had adequate dietary diversity and the most utilized food groups were *White tubers, roots and starchy vegetables*; *Pulses, nuts and seeds*; *Cereals and grains*, *Dark green leafy vegetables*, and *Fats and oils.* A larger proportion of younger women (15–29 y) were classified as anemic (20.4% versus 4.4%) and underweight (23.7% versus 5.0%) compared to older women (30–43 y). Additionally, women aged 15 to 24 years had significantly lower mean SFH-for-gestation age Z-scores than women 36–43 years (F_4, 783_ = 3.129; *p* = 0.014). Consumption of legumes nuts and seeds was associated with reduced risk of anemia while consumption of dairy products (mostly milk) was positively associated with better fetal growth. Surprisingly, low Hb level was positively associated with normal fetal growth (r_P_ = -0.133; *p* = 0.016) after 20 weeks gestation, possibly indicating normal fetal growth paralleled with physiologically necessary hemodilution.

**Conclusions:**

Sub-optimal dietary patterns, characterized by limited dietary diversity and low protein intake, are likely to compromise maternal nutrition and fetal growth in limited resource settings. Improving pregnant women’s access to cheaper but nutrient-dense protein sources such as pulses, nuts and dairy products (mostly milk) has potential to improve women’s nutritional status and enhance fetal growth.

## Background

The availability of nutrients to the fetus is known to result from associations among maternal food intake, the availability of nutrients in maternal blood, and the efficiency of the placenta in transporting nutrients to fetal circulation [1]. Hence, maternal undernutrition remains a major factor influencing fetal growth and survival, especially in limited resource setting where food insecurity remains a concern. Insufficient caloric and micronutrient intake is also associated with maternal underweight and has been associated with maternal fat losses and fetal growth restriction [2]. Although there are adaptive mechanisms that favor improved efficiency in the transfer of substrates to fetus and/or efficiency in substrate utilization when maternal nutriture is compromised, sustained nutrient deficiency has been reported to impair placenta development, placenta function, and fetal growth [1]. Severe and chronic maternal undernutrition is more specifically associated with placenta insufficiency during early pregnancy and fetal growth restriction in late pregnancy [3]. Additionally, investigations on maternal-fetal nutrient transfers indicate that chronic undernutrition may induce fetal hypoglycemia and the associated fetal gluconeogenesis which leads to fetal growth restriction [3], and increased risk of non-communicable diseases later in life [3, 4]. Hence, more studies are needed to improve understanding the dietary intake and nutritional status of pregnant women in different contexts.

It is well recognized that healthy pre-pregnancy weight (normal body mass index or BMI), consumption of a variety of foods, appropriate vitamin and mineral supplementation, and appropriate weight gain are among the essential practices for healthy maternal and fetal pregnancy outcomes. Women entering pregnancy with normal BMI usually do not need extra calories during the first trimester; however, pregnant women need an extra caloric intake of 340 during the second trimester and 452 kcal in the third trimesters to meet the growing fetal demands [5]. The problem is that many women in developing countries/Sub-Saharan Africa do not enter pregnancy with normal weight and most have limited dietary intake and high workloads which puts them at higher risk of caloric and nutrient deficits and the associated maternal undernutrition and/or suboptimal pregnancy weight gain patterns. A recent review of studies on maternal malnutrition during pregnancy indicates that the prevalence of undernutrition has been steadily increasing in Africa and was estimated at 23.5%, which makes it a problem of major public health concern [6]. The current study sought to characterize the associations between dietary intake and pregnant women’s nutritional status because Uganda is among the countries that has also documented maternal underweight associated with a higher likelihood of having low birth weight babies [7], suboptimal dietary intake among women of reproductive age [8], and consistent reports of maternal underweight and anemia in national surveys [9, 10].

In general, when women adhere to diverse diets, then they are likely to meet the increased energy and nutrient requirements given the fact that physiological changes during pregnancy result in increased nutrient absorption and improved efficiency in nutrient utilization [5]. Limited dietary diversity is associated with increased risk of preterm births, low birthweight, and maternal anemia [11–15]. The major problem in Sub-Saharan Africa is that most pregnant women chronically adhere to diets that are limited in both calories and food variety, yet most also engage in high physical activity (mostly heavy manual workloads) which significantly increases their caloric and nutrient needs. Notably, one study reported that more than 90% of pregnant women that were documented to have limited dietary diversity were also more likely to have inadequate intake of calcium, iron, zinc, riboflavin, folate, and vitamin A in both preharvest and postharvest seasons [16]. This suggests chronic limited dietary intake and the associated chronic maternal undernutrition. Hence, it is not surprising that limited dietary diversity during pregnancy has been associated with low birthweight and underweight among infants [12, 13, 17, 18]. The question is what are the specific dietary factors that need to be improved in order to improve maternal nutrition and pregnancy outcomes. The current study sought to contribute to improved understanding of dietary factors associated with maternal nutrition and fetal growth in a less-resourced, rural population in Uganda.

Besides the limited dietary diversity, the other risk factor to maternal undernutrition and the associated poor pregnancy outcomes is adhering to strict vegetarian diets. Populations that are food insecure and those that adhere to strict vegetarian diets are at increased risk of protein, energy and micronutrient deficiencies which can impair maternal nutritional status and restrict fetal growth. Hence, it has been recommended that adult women that adhere to vegetarian diets consisting of dairy products, pulses, nuts and seeds as the main protein sources should increase their dietary intake of protein from the recommended 0.8 g protein per kg body weight by 20% with a higher limit of 1 g protein per kg body weight [19]. However, this may be difficult to attain especially among populations that mainly subsist on their own food production and have limited food acquisitions from markets [20]. Since inadequate dietary patterns among women of reproductive age have been reported in dietary assessments conducted in rural Uganda [8, 21] the current study was partly designed to determine the extent to which the dietary intake of free-living, healthy pregnant women in rural areas (mostly subsistence farmers) in mid-western Uganda is associated with maternal nutritional status and fetal growth. It was hypothesized that maternal dietary intake is positively associated with maternal nutritional status and fetal growth both in early and late pregnancy.

## Methods

**Fig. 1:**
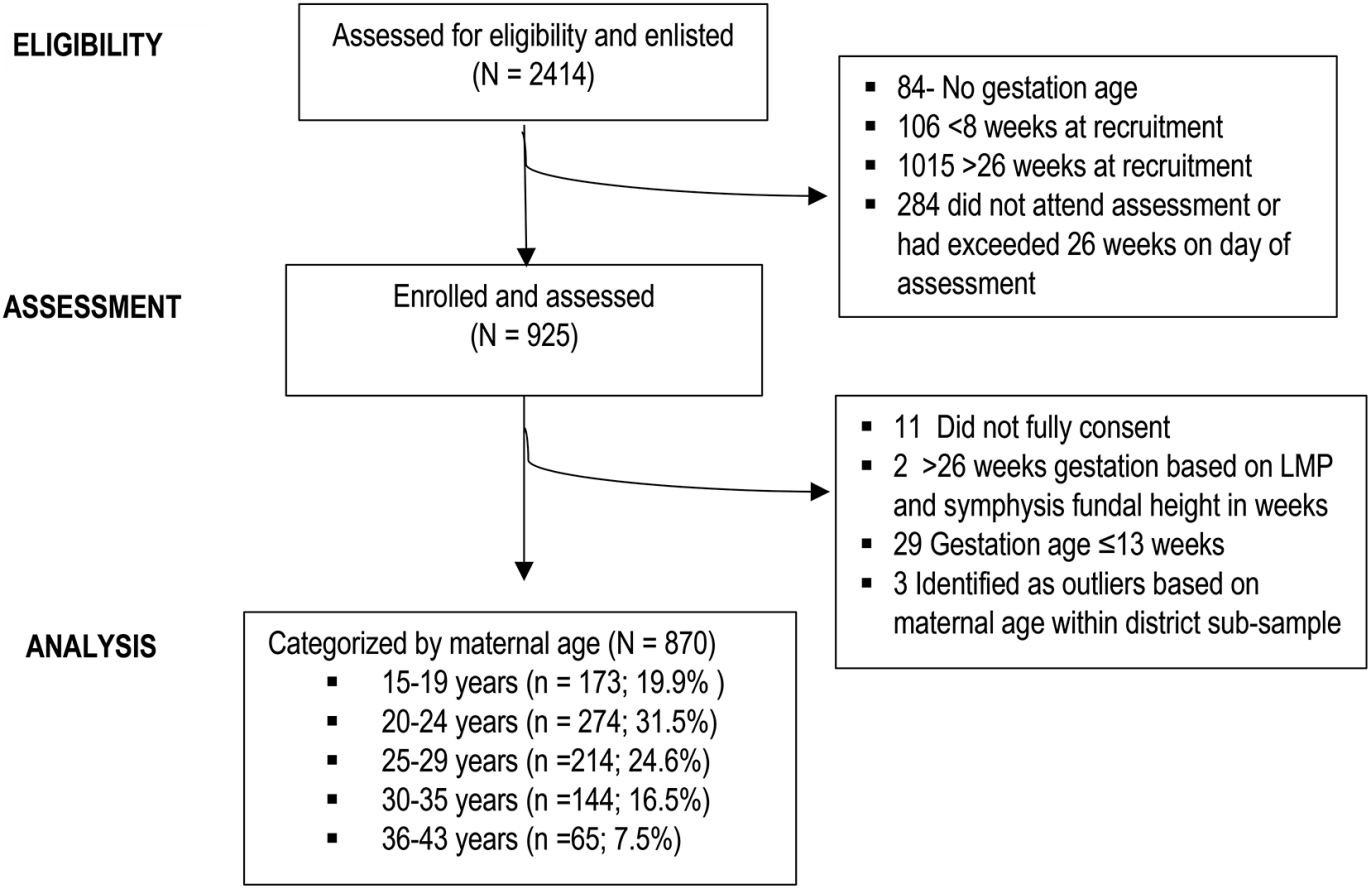
Participant selection process

This was a cross-sectional survey conducted in five districts of Kabarole, Kamwenge, Kasese, Kibale, and Kyenjojo in mid-western Uganda in August 2013. The survey targeted women of reproductive age (15–49 years), were 14 to 26 weeks of pregnancy, and resided within walking distance of ten [10] Health Centre III units (HC III) within communities with comparable socio-economic parameters. Two HC III were purposely selected from each district based on their published estimations of expected populations of pregnant women within their catchment areas. Village Health Teams (VHTs) enlisted all women that were two to six months pregnant. After 6 weeks, women that were still eligible were invited to their respective HC III for nutrition assessments (one-day health fairs). Figure 1 shows the composition of women that were recruited and included in the survey that lasted two weeks.

The process of categorizing participants by their chronological ages (Fig. 1) was guided by findings from other research that showed associations between maternal age and child birthweight [22–24]. Since literature indicates that maternal age < 18 predicts low birthweight while high maternal age of 36 years and older predicts the risk of macrosomia [25]; in most analyses the participants were further categorized into two age groups as younger (15–29 years) and older (30–43 years).

### Assessment of dietary intake

Semi-quantitative 24-hour dietary intake data was collected from each participant by post-graduate nutrition students and nurses that had undergone a one-week training. The participants’ dietary intake was quantified using the women’s dietary diversity score (WDDS) as recommended by the FAO [26]. Given the increased needs for protein and nutrient-dense foods, women’s dietary diversity was also weighted using the food consumption scores [27] to determine diet quality. The weights used were 4 for *Dairy products*, *Flesh meats*, *Fish*; *Organ meats*; and *Eggs*; 3 for *Pulses and nuts*; 2 for *Vitamin A-rich fruits and vegetables*, *Dark-green leafy vegetables*, and *Starchy staples*; and 1 for *Other fruits and vegetables*. Sugar and oil were not quantified and thus were not included in the analyses. Dietary adequacy was defined as poor when total food consumption score was 0–12, borderline at 21.5 to 35, and acceptable if > 35 [27].

**Maternal nutritional status** was assessed by measuring hemoglobin (Hb) level and mid-upper arm circumference (MUAC). Maternal capillary blood was collected from finger sticks and Hb was quantified using Hemocue hemoglobin HB 301 photometers (Hemocue AB, Angelholm Sweden) and the measured values were adjusted for altitude following the WHO guidelines [28]. Since all participants were in the second trimester, a cutoff of < 105 g/L was then used to classify women as anemic. Absolute adjusted Hb values were further used to categorize participants based on level of risk of anemia whereby < 65 g/L was considered severely anemic; 65–94.9 g/L as moderately anemic; 95–104 g/L as mildly anemic, and 105 g/L or higher as not anemic.

**Mid-upper arm circumference (MUAC**) was assessed from each woman by measuring the less dominant arm using a SECA 212 measuring tape as described by Grant and DeHoog [29]. Since there were no universally agreed MUAC cut-offs to characterize the level of malnutrition among pregnant women, a cut-off of 25 cm was used to identify participants that were malnourished (having low MUAC).

**Fetal growth** was determined using a combination of factors. First, gestation age in weeks (wGA) was estimated from each participant’s recall of the first day of the last menstrual period (LMP). Then each participant’s supine fundal height (from upper border of symphysis pubis to uterine fundus) was measured using the finger breadth technique to confirm the participant’s gestation age estimated using the reported LMP. Supine fundal height was then measured twice with a non-stretch metric measuring tape and measurements were recorded in centimeters as recommended by Belizan and colleagues [30] for all participants that met the eligibility criteria. The estimated GA (weeks) and measured fundal heights (cm) were then used to calculate the symphysis fundal height-for gestation age Z-scores (SFH-Z) using INTERGROWTH-21st Excel-based symphysis fundal height calculator [31, 32]. Fetal size was estimated using cut-offs of <-4.0 as severely small-; -4 - -2.01 as small-, and > 4 as large-for-gestation age.

Statistical methods included exploratory analyses to compare participants grouped by maternal age and gestation age. The primary indicators (mean Hb level, MUAC, and SFH) were evaluated against fixed participants’ factors such as maternal age and by time-varying covariates such as gestation age and women’s dietary diversity score (WDDS). The mean difference in Hb levels and MUAC across maternal age groups and gestation age were estimated using ANOVA. Pearson’s correlations and chi-square tests were used to assess associations between maternal dietary intake and nutritional status (Hb and MUAC) with fetal growth (SFH-Z).

## Results

### Characteristics of study participants

WHO recommends that health systems should have contact with pregnant women at 13 weeks gestation for pregnant women to start intermittent prophylactic antimalarial treatment as early as possible in the second trimester [33]. Although the current study was conducted in malaria endemic areas, 40.6% of the participant had not received any antenatal care (ANC) contact at 14–26 weeks gestation (see Table 1). Relatedly, only 51.6% reported regular use of mosquito nets and less than half (48.9%) had slept under an insecticide treated mosquito net during the night preceding the survey.

**Table 1:** Characteristics of the study participants

Characteristics	^1^Distribution of participants by age group				
	15–19 years (*n* = 173)	20–24 years (*n* = 274)	25–29 years *n* = 214)	30–35 years (*n* = 144)	36–43 years (*n* = 65)
Mean maternal age + SD	18.6 + 0.78	22.1 + 1.53	27.0 + 1.48	32.3 + 1.93	37.7 + 1.89
Mean gestation age (wGA) ± SD	20.0 ± 3.4	20.1 ± 3.5	20.3 ± 3.4	19.5 ± 3.6	20.1 ± 3.6
**Blood pressure (mm Hg)**:					
Systolic BP (mean ± SD) [95%CI]	110.9 ± 15.0^**a**^ [108.5-113.3]	107.7 ± 12.3 ^**ab**^ [106.2-109.2]	108.1 ± 13.1 ^**ab**^ [106.2-109.9]	107.7 ± 15.6^**ab**^ [105.1-110.4]	102.5 ± 10.9 ^**b**^ [102.5-108.1]
Diastolic BP (mean ± SD) [95%CI]	66.9 ± 10.0 [65.3–69.0]	66.4 ± 10.3 [65.1–67.6]	66.7 ± 8.7 [65.5–68.0]	64.4 ± 10.6 [62.6–66.2]	65.1 ± 9.6 [62.6–67.6]
***BP diagnosis criteria (%)***:					
Hypertensive (> 130/80 mm Hg)	13.7	8.1	4.6	9.0	8.2
Normotensive	74.5^a^	80.7 ^a^	88.7 ^b^	74.6 ^a^	78.7 ^a^
Hypotensive (< 90/60 mm Hg)	11.8	11.2	6.7	16.4	13.1
***ANC contacts***:					
Mean ANC visits ± SD)	0.87 ± 0.88	1.00 ± 0.93	0.82 ± 0.87	0.85 ± 0.89	0.67 ± 0.91
***% with specified ANC visits***:					
None (*n* = 353)	39.8	36.4	44.3	41.3	56.2
One (*n* = 297)	38.6	33.1	34.0	38.4	26.6
Two (*n* = 159)	17.0	24.2	17.5	15.2	10.9
Three (*n* = 43)	4.1	6.3	4.2	4.3	6.2
Four (*n* = 2)	0.1	0	0	0.1	0
%Slept under ITN in last 24 h	37.7^a^	48.7 ^b^	58.4 ^c^	51.9 ^c^	42.9 ^ab^
%Fell ill in last 14 days	31.0^a^	48.7 ^b^	45.7 ^b^	53.2 ^b^	47.6 ^b^
**% Experienced pregnancy-related complications frequently**:					
Headaches	24.5^a^	35.2 ^b^	34.0 ^ab^	34.5 ^ab^	50.8 ^c^
Heart palpitations	28.3^a^	37.4 ^bc^	33.3 ^ac^	44.1 ^bd^	55.6 ^d^
Persistent tiredness	27.7^a^	26.4 ^a^	27.4 ^a^	34.5 ^ab^	41.3 ^b^
Body weakness	25.6^a^	27.9 ^a^	34.4 ^ab^	34.5 ^ab^	44.4 ^b^
Intermittent dizziness	29.5	31.9	35.5	40.8	55.6
Difficulties with personal care	7.5	7.7	7.5	9.9	11.1
Blurred vision	6.4	3.7	6.1	7.7	7.9
Coldness of hands and feet	4.1^a^	7.0 ^ab^	5.7 ^ab^	10.6 ^b^	9.5 ^ab^

^1^Different superscript of alphabetic letters in same row indicate statistically significant differences across maternal age groups

Although the current study targeted healthy pregnant women; 45.1% reported being ill in the past two weeks preceding the assessment. Among those that reported being ill, the majority had fever or malaria (55.6%) and lower abdominal pains (44.7%). Other reported illnesses were acute respiratory infections (7.4%), headaches (7.6%), urinary tract infections (3.7%), heart palpitations (2.3%), and diarrheal infections (2.1%). Pregnancy-related complications that are considered signs of poor pregnancy outcomes that were frequently experienced included heart palpitations (37.0%), intermitted dizziness (35.5%), frequent headaches (34.0%), body weakness (31.4%), persistent tiredness (29.3%), and reported difficulties in personal care and performing daily activities of living or DALs (8.2%).

### Maternal dietary intake

**Fig. 2:**
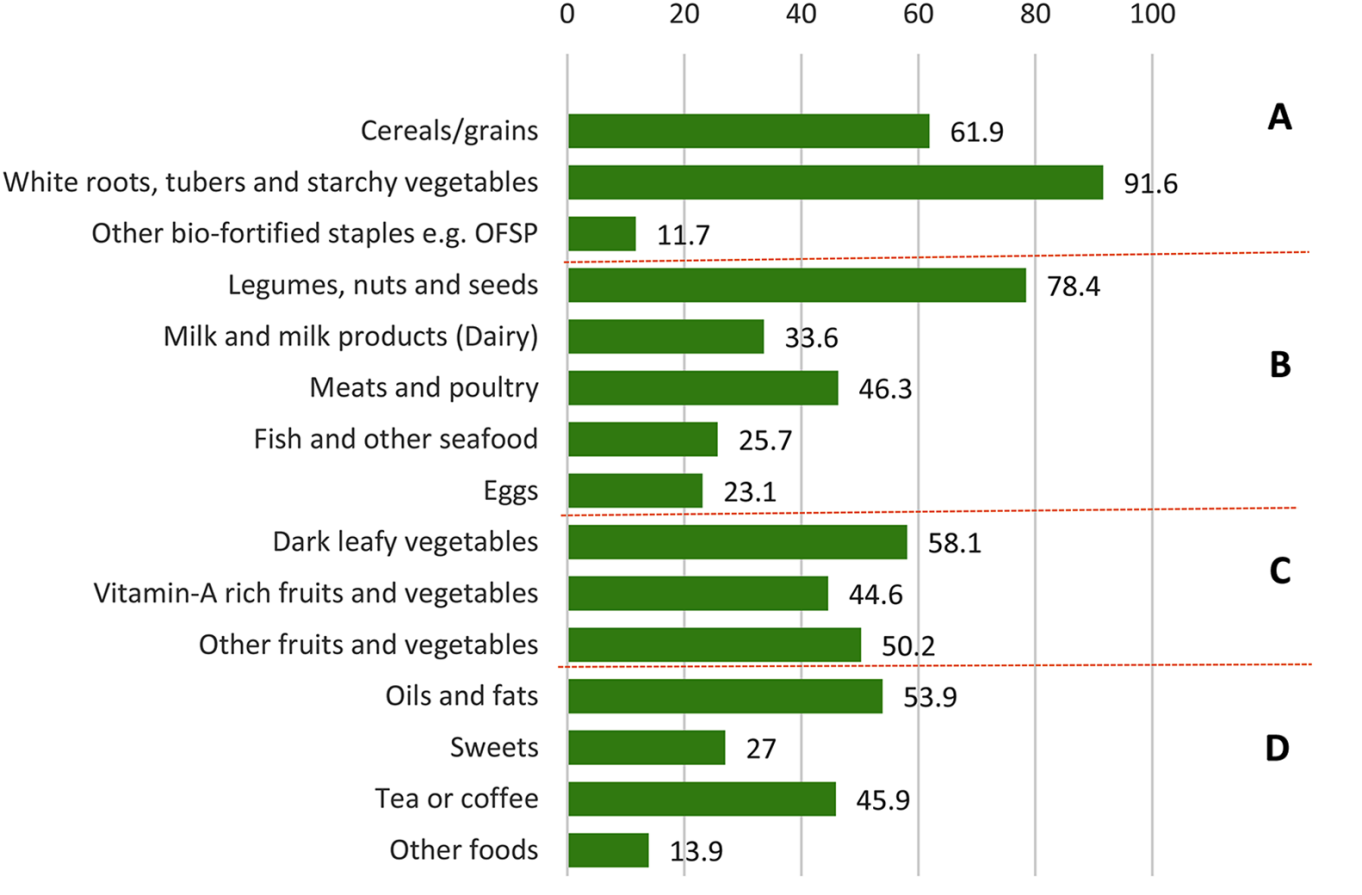
Proportion of participants that consumed foods from each group. **A** = Starchy staples; **B** = Protein-rich foods; **C** = Fruits and vegetables; **D** = Spices and condiments

Overall, the participants’ diets had limited dietary diversity. More than one third of participants had ≤ 3 food groups and almost three quarters (74.4%) had consumed ≤ 5 food groups, which indicates adherence to nutritionally inadequate diets. The most utilized food groups were *White tubers, roots and starchy vegetables*; *Pulses, nuts and seeds*; *Cereals and grains*, *Dark green leafy vegetables*, and *Fats and oils* (see Fig. 2). Only 11.7% included bio-fortified foods such as orange-fleshed sweet potatoes. Despite the increased protein needs in pregnancy, the participants’ intake of all animal-source foods was also very limited. Even dairy products (mostly milk), which are the least costly and culturally accepted protein-rich food in the study area, were consumed by only one third of participants.

Based on food consumption scores [27], none of the participants assessed met the requirements for an acceptable diet. Out of the 864 women that gave complete dietary data, 91.9% had poor diets and only 8.1% were classified as having diets of borderline quality; and this is not surprising since participants that had consumed three groups or less had only included starchy staples, legumes (beans), and leafy vegetables.

### Maternal nutritional status

One quarter of the participants (26.0%) were classified as anemic (Hb < 105 g/L) after adjusting for gestation age and altitude [34], 28.6% were underweight (MUAC < 25 cm), and 5.4% were severely underweight (MUAC < 23 cm). As shown in Table 2 women 15–19 years had a significantly lower mean MUAC than other age groups (F_4, 858_ = 10.14, *p* = 0.000) and MUAC remained positively associated with maternal age after controlling for gestation age (r _(844)_ = 0.217, *p* = 0.000), even though a significantly lower proportion of younger women reported being ill in the 2 weeks preceding the assessment (Table 2).

**Table 2:** Prevalence of maternal undernutrition and low fetal growth rate

Characteristics	^1^Distribution of participants by age group				
	**15–19 years** (*n* = 173)	**20–24 years** (*n* = 274)	**25–29 years***n* = 214)	**30–35 years** (*n* = 144)	**36–43 years** (*n* = 65)
Mean Adjusted Hb (g/L) ± SD [95% CI] (*N* = 826)	113.6 ± 17.0 [111.0-116.3]	112.1 ± 17.1 [110.0-114.2]	113.1 ± 17.3 [110.7–115.5]	116.9 ± 15.7 [114.2 -119.5]	113.3 ± 16.6 [109.0-117.5]
***Severity of anemia (%)***:					
**Mildly anemic**	**15.4** (25)^**ab**^	**17.0** (44)^**b**^	**13.1** (27)^**ab**^	**9.4** 913)^**a**^	**9.8** (6)^**ab**^
Moderately anemic	12.3 (20)	11.2 (29)	12.1 (25)	8.0 (11)	16.4 (10)
Severely anemic	0.6 (1)	0.8 (2)	1.0 (2)	0	0
***Maternal underweight***:					
Mean MUAC (cm) ± SD [95%CI]	25.6 ± 1.9^**a**^ [25.3–25.9]	26.3 ± 2.3^**ab**^ [26.0-26.5]	26.6 ± 2.7^**bc**^ [26.2–27.0]	27.2 ± 3.0^**c**^ [26.7–27.7]	27.2 ± 2.8^**c**^ [26.6–27.9]
%Low MUAC (< 25 cm)	38.5 (65)^a^	27.3 (74)^ab^	29.0 (61)^ab^	21.7 (31)^b^	21.5 (14)^b^
Mean hip circumference [95%CI]	92.0 ± 5.4^**a**^ [91.2–92.8]	92.8 ± 5.6^**a**^ [92.1–93.5]	93.1 ± 7.1^**a**^ [92.1–94.0]	94.0 ± 7.1^**ab**^ [92.8–95.1]	95.4 ± 7.0^**b**^ [93.7–97.1]
Mean head circumference [95%CI]	54.0 ± 1.6 [53.8–54.3]	54.2 ± 1.9 [54.0-54.5]	54.4 ± 3.6 [53.9–54.9]	54.1 ± 1.7 [53.8–54.4]	54.4 ± 1.8 [54.9–54.8]
***Fetal growth***:					
^2^Mean SFH Z-score ± SD [95%CI] *N* = 788	-1.43 ± 1.25^**a**^ [-1.63, -1.23]	-1.28 ± 1.36^**a**^ [-1.46, -1.11]	-1.19 ± 1.39^**ab**^ [-1.39, -1.0]	-1.14 ± 1.38^**ab**^ [-1.39, -0.90]	-0.72 ± 1.57^**b**^ [-1.13, -0.30]

^1^Varying superscripts of alphabetic letters in same row indicate statistically significant differences across maternal age groups

^2^Fundal height z-scores were computed for participants 16–26 weeks gestation only

**Fig. 3:**
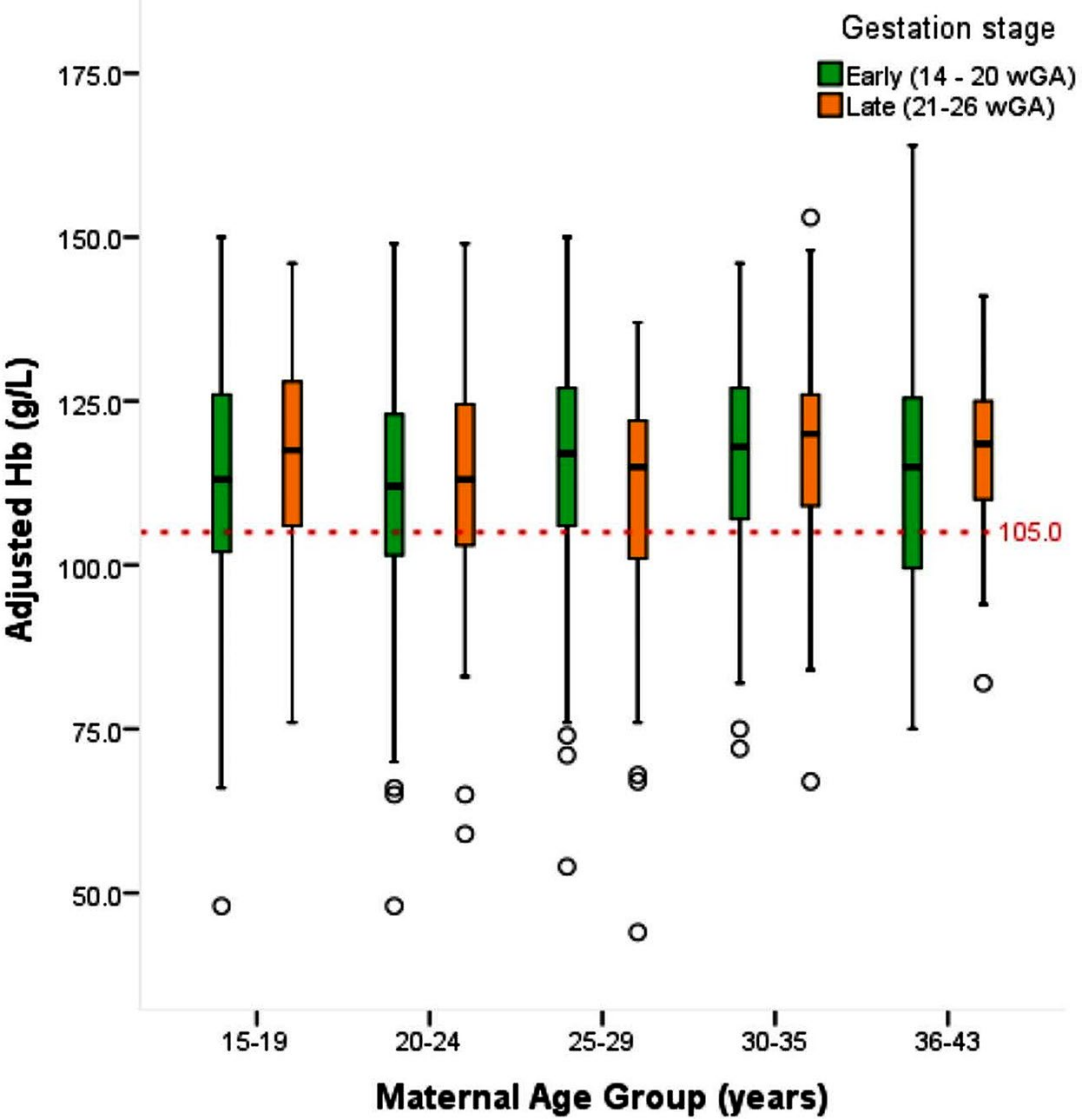
Hemoglobin levels across maternal age groups by stage of gestation

Maternal age and gestation stage were not related to Hb level (Fig. 3). However, a significantly larger proportion of younger women (15–29 y) were categorized as anemic when compared to those aged 30–43 y (27.1% versus 19.1%; X^2^_(1,826)_ = 5.154; *p* = 0.023). Further analyses revealed that the associations between maternal age and risk of anemia were more pronounced at 21 to 26 wGA (the maternal catabolic phase) whereby almost twice as many young women were anemic compared to older women (28.4% versus 15.9%; X^2^_1,826_ = 4.417; *p* = 0.036).

### Fetal growth

**Fig. 4:**
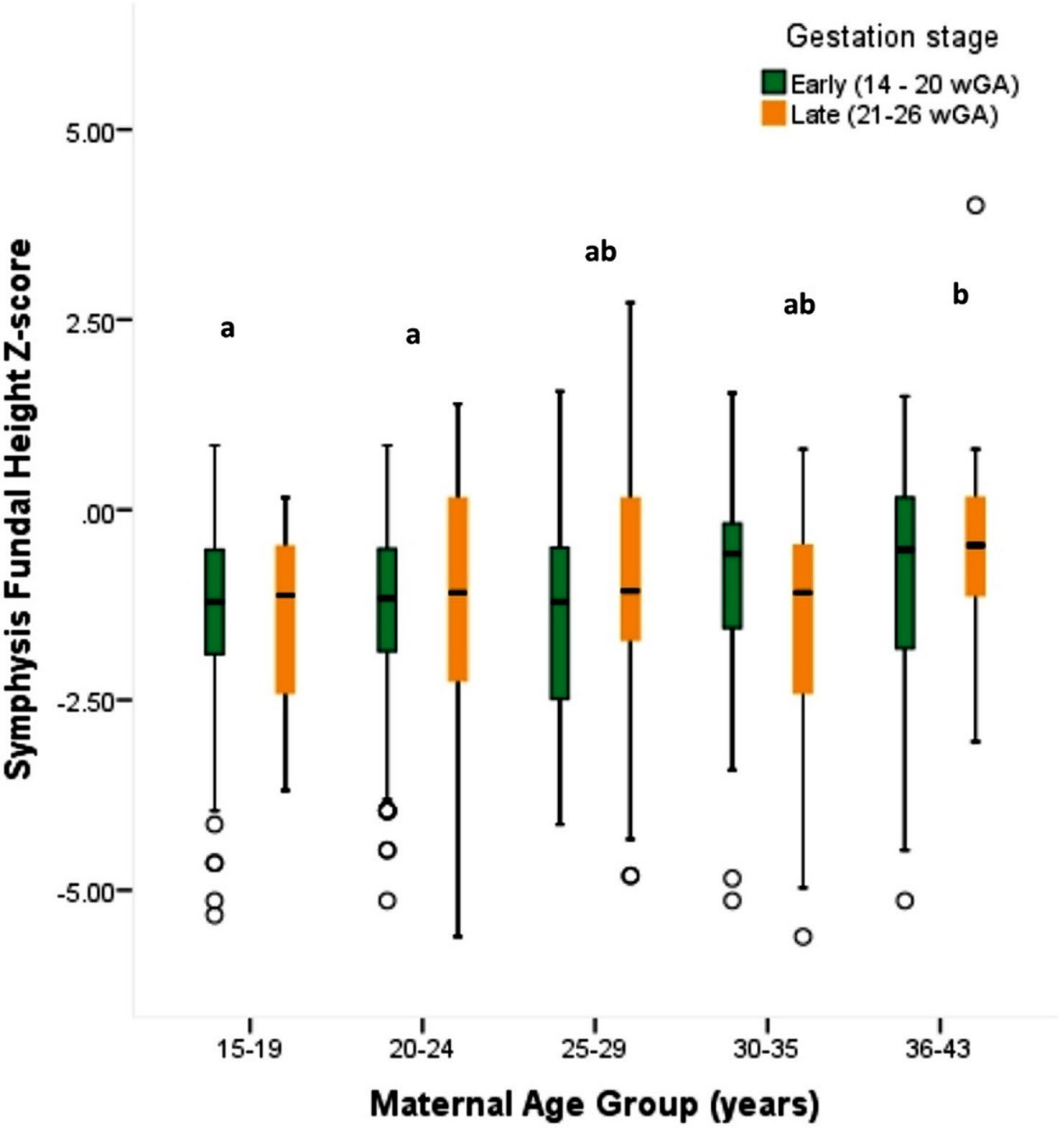
Fetal growth (SFH-Z) by maternal age group and gestation stage. Superscripts that vary denotes significant differences in mean SFH-Z

In resource-constrained contexts where access to ultrasound scanning is limited, fundal height is considered a reliable indicator of fetal growth, especially when assessed by qualified health workers [35, 36]. In the current study, overall mean symphysis fundal height (SFH) z-score (Mean = -1.23 ± 1.38) was low but within the normal fetal growth trajectory. Based on Intergrowth chart categorizations, 15.9% of participants 16–26 weeks pregnant had estimated small-for-gestation age pregnancies and 4.7% of these were classified as severely small (<-4 z-score). Women aged 15 to 24 years had significantly lower mean SFH-for-gestation age Z-scores than women 36–43 years (F_4, 783_ = 3.129; *p* = 0.014) (see Fig. 4); which indicates that younger women had an increased risk of giving birth to low birthweight babies.

### Associations among maternal dietary intake and nutritional status with fetal size

Overall, the risk of anemia was also positively associated with maternal nutritional status indicated by absolute MUAC measurement (r_p_ = 0.077; *p* = 0.029); and this relationship remained significant after controlling for gestation age (Partial *r* = 0.078; *p* = 0.027) and total weighted dietary diversity score (Partial *r* = 0.086; *p* = 0.017). Further analyses revealed that the association between maternal Hb and MUAC was more pronounced at 14–20 wGA (r_p_ = 0.101; *p* = 0.027), especially after controlling for diet quality (Partial r_p_ = 0.114; *p* = 0.015). This can partly be explained by adequate diets supporting the anabolic processes that favor accretion of maternal tissue including arm muscle and fat (MUAC) and body proteins such as Hb during early pregnancy (0–20 wGA or the maternal anabolic phase). Both maternal MUAC and Hb level were not associated with fetal growth indicated by SFH-Z (see Table 3).

**Table 3:** Correlations among maternal dietary intake and nutritional status with fetal growth indices

Characteristic	^*1*^*Adj Hb* (*N* = 814)		*MUAC* (*N* = 814)		*SFH –Z* (*N* = 737)	
	Correlation coeff	*P*-value	Correlation coeff	*P*-value	Correlation coeff	*P*-value
*MUAC* (*N* = 814)	**0.077**	**0.029**	-	-		
*SFH –Z* (*N* = 737)	− 0.064	0.080	0.041	0.265	-	-
*Total WDDS*	0.029	0.416	− 0.021	0.548	0.042	0.244
*Variety within food groups* (*N* = 706):						
Starchy staples	− 0.056	0.139	**− 0.087**	**0.021**	0.015	0.686
Dark-green leafy vegetables	0.047	0.216	0.018	0.632	0.044	0.248
Other vitamin A-rich fruits and vegetables^1^	0.024	0.524	− 0.042	0.270	0.012	0.744
Other fruits and vegetables	0.069	0.065	0.032	0.391	− 0.010	0.800
Organ meats	− 0.022	0.567	− 0.036	0.345	0.027	0.481
Meats and fish	− 0.058	0.125	− 0.057	0.128	0.015	0.686
Eggs	− 0.013	0.726	− 0.025	0.507	0.003	0.928
Legumes/pulses, nuts, and seeds	**0.100**	**0.008**	0.012	0.749	0.035	0.353
Milk and other milk products	− 0.008	0.842	0.003	0.942	**0.080**	**0.034**

N = Reflects the total participants included in particular analysis. ^1a^ Included only those with MUAC and Hb data. ^1b^Includes only women 16 wGA for whom SFH-Z scores were calculated. ^***1c***^Includes those with complete dietary diversity data

^2^The category of *Bio-fortified foods and orange-fleshed fruits and vegetables* was not included because no one selected foods from group

In general, maternal diets were not significantly different across age groups and stage of gestation. As shown in Table 3, consuming a variety of foods from the *Pulses, nuts and seeds* group was positively correlated with high Hb indicating a reduced risk of anemia (coeff (r_p_) = 0.100; *p* = 0.008). On the contrary, consumption of a variety of *Starchy staples* (which in this context may suggest reduced access to the major staples and/or reliance on food purchases) was associated with low MUAC (coeff (r_s_) = -0.077; *p* = 026) or increased risk of maternal underweight. Although the consumption of dairy products was not related to measures of maternal nutritional status (Hb and MUAC), participants that reported including dairy products in their diets had higher symphysis fundal height measurements (coeff (r_s_) = 0.080; *p* = 0.034), which suggests better fetal growth.

## Discussion

Overall, the current study indicates sub-optimal maternal dietary patterns characterized by limited animal-source foods and emphasis on starchy staples, legumes (beans), and leafy vegetables. These dietary patterns place pregnant women at risk for undernutrition and poor pregnancy outcomes. However, overall maternal dietary intake was not directly associated with maternal nutritional status and fetal growth both in early and late pregnancy as it was hypothesized. It was maternal age (> 30 years) that was associated with better maternal nutritional status indicated by MUAC and fetal growth.

Generally, MUAC has been identified as a reliable predictor of maternal nutritional status indicated by maternal weight and BMI in different contexts [37–40]. MUAC has also been positively correlated to BMI among pregnant women at 19–21, 27–29, and 37–39 weeks of gestation [38], which indicates that MUAC is a good predictor of maternal nutritional status at different stages of pregnancy. Hence, the positive associations between low MUAC and low Hb documented in the current study indicates a high incidence of malnutrition among pregnant women in rural mid-western Uganda. This high incidence of maternal undernutrition raises a concern since maternal undernutrition is associated with increased risk of low fetal size and low birthweight [40–44]. Hence, there is need to improve reach and/or boost uptake of on-going food fortification and micronutrient supplementation interventions in Uganda to reduce anemia among resource-constrained pregnant women in similar contexts. More studies are also needed to investigate the implications of poor maternal nutritional status on fetal growth using more precise measures for estimating fetal size besides measuring fundal height [35]. The differential associations between maternal nutritional status and fetal growth at different stages of pregnancy also need in-depth investigations.

As has been observed in other studies, this research revealed younger women might be at higher risk of undernutrition and possibly poor fetal birth outcomes. Notably, younger women (15–29 years) had a higher prevalence of anemia and low MUAC at 20–26 weeks gestation. As it has been established in other studies [23], young women (especially those < 25 years old) are still growing and are more likely to experience negative impacts associated with maternal nutrient mobilization to support fetal growth. Since the human body naturally prioritizes fetal growth after 20 weeks GA, this indicates that undernourished young women may not be able to catch up on expected pregnancy weight gain in the remaining half of the pregnancy duration due to the combined high maternal energy and nutrient needs for own growth and the increasing fetal nutritional needs. Although all women have been documented to experience MUAC reductions as pregnancy advances, young women have been documented to experience more losses [45]. These findings suggest that when resources are limited, there is need to prioritize improving the nutrition of young pregnant women to improve both maternal and fetal birth outcomes. Conversely, since it has been suggested that adult women are less likely to experience pronounced changes in MUAC during the course of pregnancy [40, 43, 45, 46], which calls for screening older women with more precise tools to avoid excluding those that are undernourished from vital interventions.

Another key finding from the current study was the limited animal-source food consumption despite the increased need for high quality and adequate protein during pregnancy. The positive association between maternal dairy product consumption and fetal growth suggests that lower cost animal-source foods such as milk (which is consumed regularly) might have more potential to impact maternal nutrition and fetal growth than meats, poultry and fish products which are expensive and consumed occasionally.

Given the limited consumption of animal-source foods, the positive association between *Pulses, nuts and seeds* with Hb level is not surprising since beans constituted a major relish and the main source of iron and protein, especially in rural areas of mid-western Uganda. Conversely, the negative association between consumption of flesh meats and Hb level possibly indicates that meats were being used as an iron-rich food to prevent or manage anemia among the women that were at high risk of (or were already diagnosed with) anemia. It is important to note that the current study was conducted in the planting season for beans (a major relish and protein source that is consumed almost daily in many households in western Uganda). Hence, the positive association of Pulses and nuts with Hb might also indicate better food security since more resource-constrained households are likely to have been using leafy vegetables as relish during the study period.

One major limitation of the current study was that dietary intake was assessed from the women’s recall of their dietary practices. In general, self-reported dietary intake has been associated with potential errors of overreporting desirable foods while underreporting less-desirable foods. Overreporting tendencies are likely to explain the negative but not significant associations between consumption of flesh meats with Hb level.

In conclusion, the current study has identified the need to prioritize nutrition support for young women (< 30 years) since they exhibited a higher risk of maternal anemia, underweight, and had lower symphysis fundal height – all of which indicate a high probably of giving birth to smaller-for-gestation age babies. Although the current study suggests that younger women are at higher risk in the second half of pregnancy (> 20 wGA), it is important initiate nutrition support early in pregnancy to ensure that young women (especially the adolescents) attain and maintain adequate nutritional status to support their own growth and the increasing needs of the growing fetus. The current study also highlights the need for targeted food security interventions to address the unique dietary needs of pregnant women. Notably, it shows that improving pregnant women’s access to cheaper but nutrient-dense protein sources such as pulses, nuts, and dairy products (mostly milk) has potential to improve women’s nutritional status and enhance fetal growth. This finding is especially generalizable in contexts where animal-source foods are expensive and/or less accessible to pregnant women and those contexts where food security interventions mainly focus on promotion of increasing production of the carbohydrate-rich staple foods. Conversely, where agricultural production in being promoted, there is need to promote bio-fortified staples to enhance access of nutrient-rich foods among pregnant women.

## Data Availability

The dataset generated and analyzed for the current study is available from the corresponding author on reasonable request.

## Abbreviations

ANC: Antenatal care
BMI: Body mass index
BP: Blood pressure
Hb: Hemoglobine
HC III: Health Centre III
ITNs: Insecticide treated mosquito nets
LMP: Last menstrual period
MUAC: Mid-upper arm circumference
SFH: Symphysis fundal height
WDDS: Women’s dietary diversity scoren
wGA: Weeks gestation (weeks of pregnancy)
y: Year
